# Leaf Longevity as a Normalization Constant in Allometric Predictions of Plant Production

**DOI:** 10.1371/journal.pone.0081873

**Published:** 2013-12-03

**Authors:** Kihachiro Kikuzawa, Kenji Seiwa, Martin J. Lechowicz

**Affiliations:** 1 Laboratory of Plant Ecology, Ishikawa Prefectural University, Nonoichi, Japan; 2 Laboratory of Forest Ecology, Tohoku University, Osaki, Japan; 3 Department of Biology, McGill University, Montréal, Canada; Centre National de la Recherche Scientifique, France

## Abstract

In metabolic scaling theory the size-dependence of plant processes is described by a power function of form *Y*=*Y*_*o*_ *M* ^*θ*^ where *Y* is a characteristic such as plant productivity that changes with plant size (*M*) raised to the *θ* ^*th*^ power and *Y*_*o*_ is a normalization constant that adjusts the general relationship across environments and species. In essence, the theory considers that the value of θ arises in the size-dependent relationship between leaf area and vascular architecture that influences plant function and that *Y*_*o*_ modulates this general relationship to account for ecological and evolutionary effects on the exchange of resources between plant and environment. Enquist and colleagues have shown from first principles that *Y*_*o*_ is a function of carbon use efficiency, the carbon fraction of a plant, the area-specific carbon assimilation rate of a leaf, the laminar area of a leaf, and the mass of a leaf. We show that leaf longevity provides a functional integration of these traits that can serve as a simpler normalization in scaling plant productivity for individual species and potentially for mixed-species communities as well.

## Introduction

West, Brown and Enquist [1] proposed a general theory for plant growth and primary production founded on the idea that the architecture of vascular systems controls the scaling of leaf surface area on plant size, which in turn governs the exchange of resources between plant and environment. Their original WBE theory set out a general framework for scaling this size-dependence of plant form and function: 

$$Y=Y_{0}M^{\theta }$$ (1)

where *Y* is some characteristic such as respiration rate, leaf biomass or growth rate that changes with plant size (*M*) and *Y*_*o*_ is a normalization constant that adjusts the general relationship across environments and species. A core assumption in WBE theory is that the value of *Y*_*o*_ is determined by the metabolic demand imposed by leaves. In essence, the value of θ arises in the way that the size-dependent relationship between leaf area and vascular architecture influences plant function and *Y*_*o*_ modulates this general relationship to account for ecological and evolutionary effects on the exchange of resources between plant and environment. Metabolic scaling theory (MST; *cf*. summary review [2]) is the contemporary outcome of refinements and elaboration of this basic WBE framework and part of wider developments in the Metabolic Theory of Ecology (MTE; cf. summary review [3]). The contemporary view is that while there is a degree of interspecific variation in both *Y*_*o*_ and θ, the scaling coefficient θ for plants holds close to a global value of ¾ while the normalization *Y*_*o*_ can vary more widely as a function of traits characterizing foliar investment and function [4].

Recognizing the central role of leaf longevity in plant productivity processes, we suggested [5] leaf longevity might serve to normalize allometric predictions of plant production. This possibility is rooted in the fact that carbon assimilation by plants can be expressed as the mathematical product of three elements: the amount of foliage, the photosynthetic rate and the seasonal duration of photosynthetic activity. Leaf longevity connects instantaneous photosynthetic rate to the duration of photosynthetic activity from leaf emergence through leaf senescence [5], and provides a link between size dependent investment in total foliar biomass and the array of individual leaves in the plant canopy [6]. Use of leaf longevity as a normalization could provide a useful synthesis between theory for leaf longevity [7] and the more general metabolic scaling theory for size-dependent variation in plant form and function [2]. Here we lay out a theoretical rationale to support the prediction of primary production for individual plants and in monospecific stands using an allometric function normalized by leaf longevity and the amount of leaves, and we make a preliminary test of the predictions using what few data are available.

## Theory and Results

Plant primary production (carbon equivalent; *P*, g C yr^-1^ plant^-1^) can be expressed [5] by the following equation: 

$$P=Ā\delta M_{L}$$ (2)

Where *Ā* (g C g leaf^-1^ s^-1^) is the average instantaneous photosynthetic capacity of a single leaf over its life span from emergence (*t*=0) when the capacity is highest to senescence (*t*=*L*, leaf longevity, days), δ (s yr^-1^) is the duration of potential photosynthetic rate within a year, and *M*_*L*_ (g leaf plant^-1^) is the leaf biomass of an individual plant. Since photosynthetic rate declines linearly with time [8-10], *Ā* is expressed as

$$Ā=\frac{A(0)+A(L)}{2}$$ (3)

Theory for leaf longevity [8] incorporates a linear decrease in photosynthetic rate with time:

$$p(t)=a(1-t/L_{p})$$ (4)

where *p*(*t*) (g C g leaf^-1^ day^-1^) is the daily photosynthetic rate at time *t*, *a* (g C g leaf^-1^ day^-1^) is the daily photosynthetic rate at time zero and *a/L*_*p*_ (g C g leaf^-1^ day^-2^) is the rate of decline in photosynthetic capacity with time; *L*_*p*_ (days) is the leaf age at which the photosynthetic rate becomes zero -- the potential leaf longevity [11]. Instantaneous photosynthetic capacity is assumed to be expressed similarly as:

$$A(t)=A(0)(1-t/L_{p})$$ (5)

Therefore, from equations (3) and (5), *Ā* can be taken as: 

$$Ā=A(0)(1-L/2L_{p})$$ (6)

The duration of potential photosynthetic rate within a year, δ in equation (2), can be decomposed into two components: a) duration within a day or mean labor time *m* (s day^-1^) and b) duration within a year, or favorable period, *f* (days year^-1^[5,12]):

δ=mf (7)

Optimum leaf longevity is obtained by maximizing the photosynthetic gain per unit time [8] using equation (5) as:

$$t_{opt_{}}={\left(\frac{2L_{p}C}{a}\right)}^{1/2}$$ (8)

where *t*_*opt*_ (days) is the optimal leaf longevity to maximize marginal gain (gain per unit time), *a* = *m* *A*(*0*), *C* (g leaf g carbon^-1^) is taken the invariant construction cost of a leaf. Putting actual leaf longevity equal to optimum leaf longevity, we obtain mean labor time from equation (8) as:

$$m=\frac{2L_{p}C}{L^{2}A(0)}$$ (9)

The leaf biomass on an individual plant, *M*_*L*_ in equation (2), scales as the *θ* ^th^ power of total plant mass *M* (g plant^-1^), and a normalization constant β as shown in equation (1). 

$$M_{L}=\beta M^{\theta }$$ (10)

where both the normalization constant β and the scaling exponent θ are taken as invariant [2]. Substitution of equations (6), (7), (9) and (10) into (2) gives the following allometric relation for the surplus production by the *j* ^th^ plant of *i* ^th^ species.

$$P_{i,j}=\frac{fC}{L_{i}}\left[\frac{2L_{pi}}{L_{i}}-1\right]\beta M_{j}^{\theta }$$ (11)

It should be noted that the favorable period (f) in a locality will vary both because of species-specific differences in functional responses to factors limiting production and because of microenvironmental effects on individual plants [5,7]. Assuming that the variance component in *f* across localities substantially exceeds that within localities, then as a first approximation we can consider *f* constant in a locality. The potential (*L*_*p*_) and realized (*L*) leaf longevities on the other hand are known to vary substantially among species within a locality [7]. We therefore can take the allometric exponent (*θ*) scaling leaf biomass on plant size as ¾ and consider that within a locality the normalization constant β will be adjusted primarily by species-specific variation in leaf longevity. This is a parsimonious alternative to earlier approaches to trait-based scaling of production on plant size that involve more parameters [2,4]. 

Equation (11) shows that production is determined by only three component factors. The first is leaf longevity. Production should be inversely related to leaf longevity, a prediction supported by several empirical studies [13,14]. The second factor is the ratio of potential and realized leaf longevity. It is usually the case that realized leaf longevity is less than the potential so the value in the bracket of equation (11) generally takes a value greater than 1. When this term is unity, it implies that the amount of leaves produced by a plant are the same from one cohort to another and when the term is greater than 1, the plant produces more leaves (or branches, stems, roots, flowers, fruits) using excess carbon. Finally, the third term is plant size. The greater the plant size, the more the leaf biomass, and thus the greater the production. The relationship between plant mass and production is allometric, mainly because of the accumulation of nonfunctional conducting tissues in the stems of woody perennial species. The progression of self-shading also contributes to the allometric relationship between *P* and leaf biomass [15].

### A further simplification

**Figure 1:**
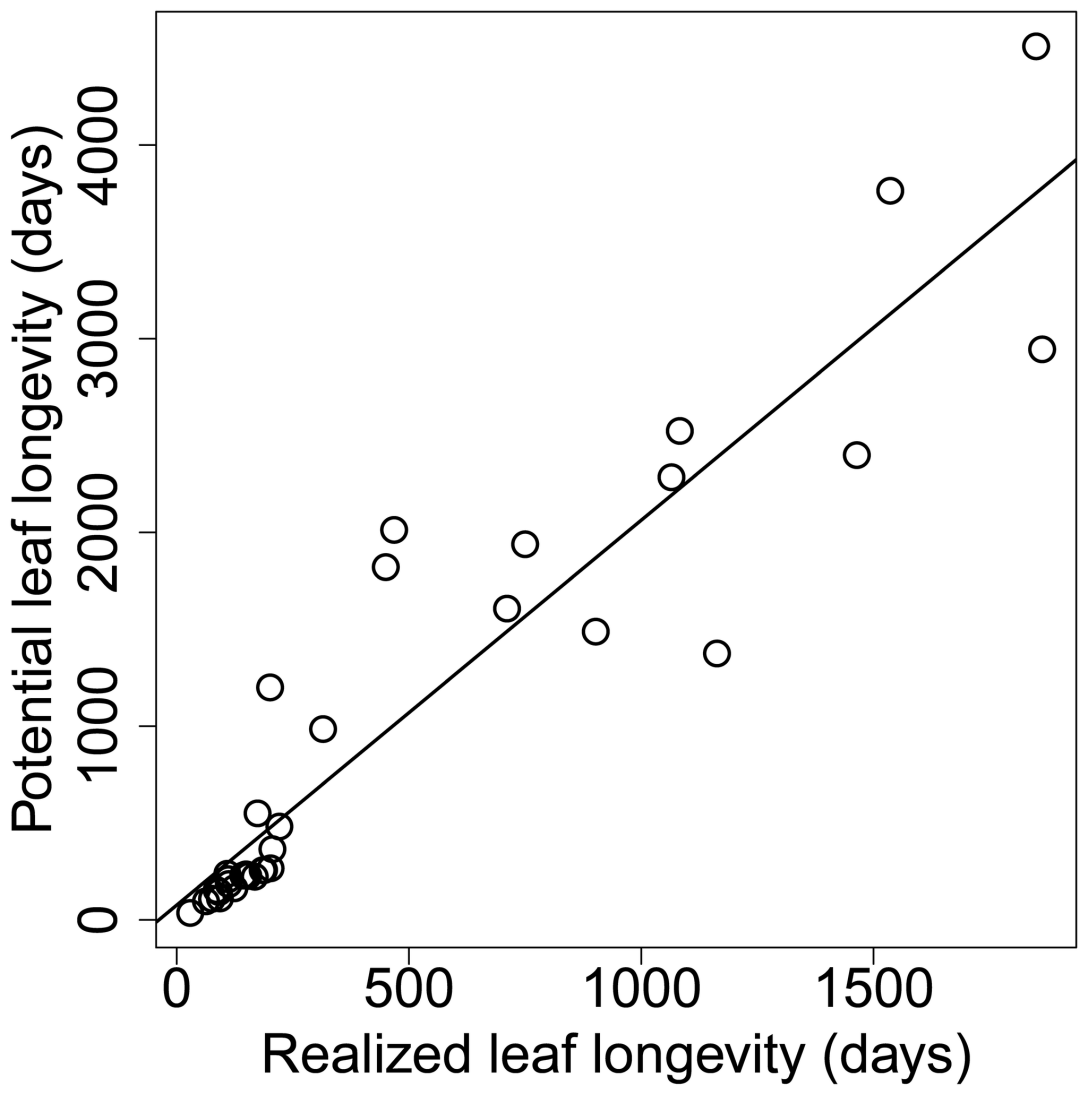
The relationship between potential leaf longevity (*L_p_*) and actual leaf longevity (*L*): *L*_*p*_ =2.0 *L*+94 days (r = 0.93). Potential leaf longevity was estimated for each leaf or as an average value for each species at the leaf age when the maximum photosynthetic rate declines to zero. Maximum photosynthetic rates were measured repeatedly for each leaf [6], or for some leaves of the same species [9] and regressed against leaf age to estimate potential leaf longevity for 34 cases representing 29 species; we also used results from one previously published [23] relationship between *L*_*p*_ and *L*.

The biomass (*M*) of single plant can be readily estimated allometrically using stem diameter in equation (10). Leaf longevity has been measured in many plant species [7,16], but the estimation of potential leaf longevity requires repeated measurements of photosynthetic rate of the same leaf over its lifespan. Hence parameter *L*_*p*_ is not easily determined and data are scarce. We examined the ratio *L*_*p*_ */L* in 34 species-year-site combinations of potential and realized leaf longevity and found that the ratio ranged from 1.18 to 5.97, with an average of 2.08 (std dev1.03). Potential leaf longevity was linearly correlated with actual leaf longevity (Figure 1) so for simplicity we can set *L*_*p*_ /*L*=2 and rewrite equation (11) as:

$$P_{i,j}=3\frac{fC}{L_{i}}\beta M_{j}^{\theta }$$ (12)

This suggests that primary production is affected by the amount of leaves and the inverse of their longevity. If we compare the production of plants of similar size at the same site, the production is determined only by the inverse of leaf longevity.

### Relative growth rate

**Figure 2:**
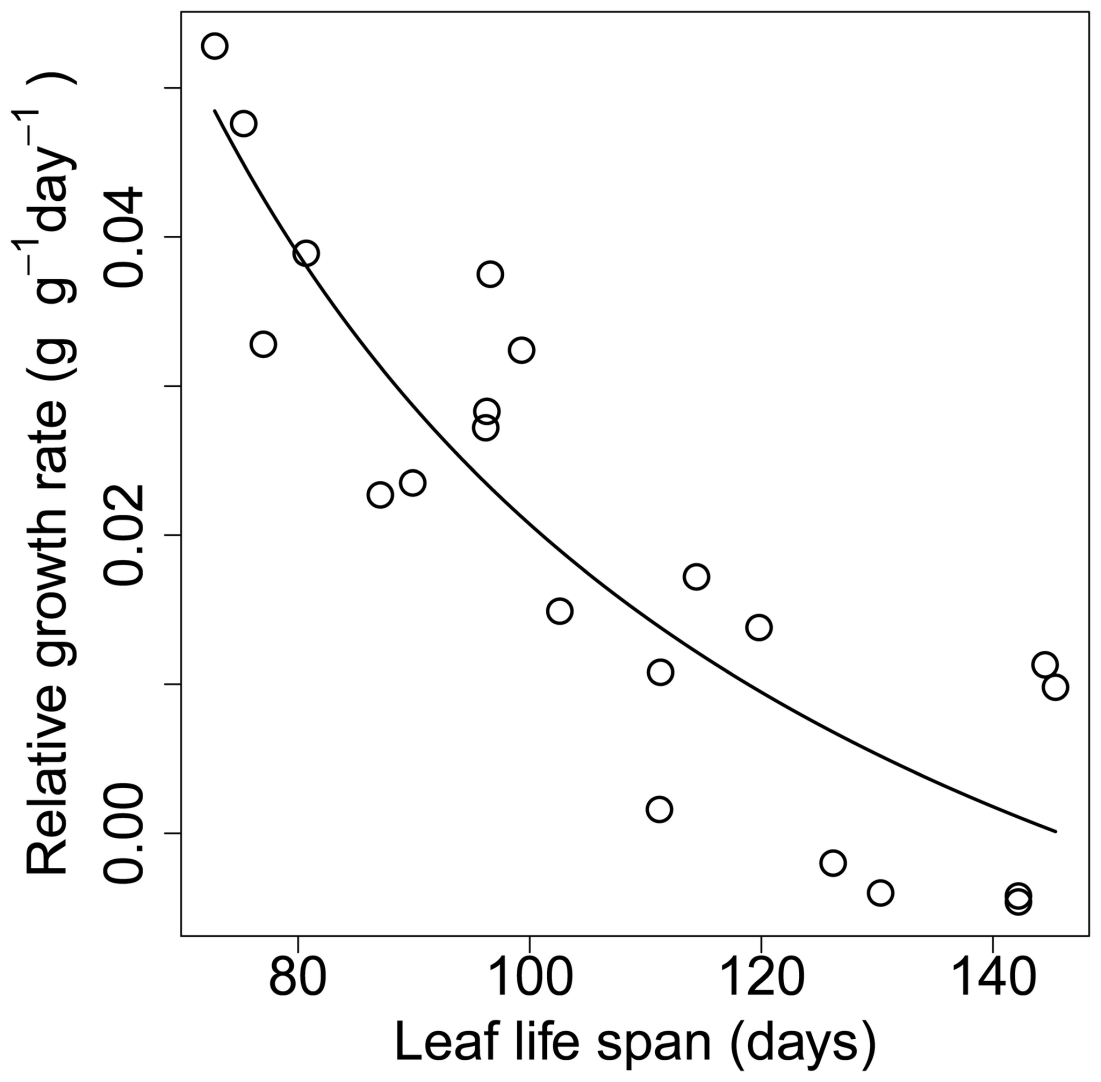
The clearly inverse relationship between seedling relative growth rate (RGR) and leaf longevity in *Betula platyphylla japonica, Cercidiphyllum japonicum, Acer mono* and *Quercus mongolica* *grosseserrata*: RGR = 5.1/(*L*-14.4)-0.04 (g g^-1^ day^-1^, day), r^2^ = 0.79. The data points are averages for five to ten seedlings of each species sown in large gap, small gap and forest understory habitats in a deciduous broad-leaved forest in Hokkaido, Japan. Leaf longevities are based on weekly census from seedling emergence to the end of the first growing season. Relative growth rates are based on dry weight at harvest in early September. Based on data in and redrawn from [14].

Relative growth rate (RGR, g C g C^-1^ year^-1^), the growth per unit time relative to plant size (*dM*/*dt*/*M*), is an important determinant of plant productivity. If we assume that a fixed ratio (γ, [g C yr^-1^ plant^-1^ ]/[g C yr^-1^ plant^-1^] ) of production is allocated to incrementing plant size (body mass, *M*) then $\frac{dM}{dt}=\gamma P=3\gamma \frac{fC}{L}\beta M^{\theta }$ and *RGR* is $\frac{1}{M}\frac{dM}{dt}$ or 

$$RGR=3\gamma \frac{fC}{L}\beta M^{\theta -1}$$ (13)

where *γ* is a species-specific constant and *f* is constant for a given site. Hence in an intraspecific comparison, *RGR* will be determined only by plant mass. Since *θ* can be taken as ¾, *RGR* scales as -1/4 of plant mass and therefore should be governed chiefly by variation in leaf longevity. In interspecific comparisons, however, leaf longevity, *β* and *γ* will also contribute the variation in *RGR*. If first-year seedlings are compared, *γ* is invariant among species since plant size at the end of the first growing season is determined by only current year production. The effect of *θ* will also disappear in equation (13) in the case of seedlings, since *θ* takes a value near unity in small plants. The anticipated negative relationship between *RGR* and leaf longevity [2,14] has been documented in four deciduous broad leaved seedlings (Figure 2). 

### Leaf Lifetime Gain

The lifetime gain of a leaf (*G*_*L*_, g C g leaf^-1^ leaf longevity^-1^), the amount of carbon that a single unit of leaf assimilates from emergence to senescence, can be expressed as

$$G_{L}=ĀmL_{f}$$ (14)

Where *L*_*f*_ (days) is the functional leaf longevity, which is the number of actual photosynthetic days within the leaf lifetime. Equation (2) can be rewritten using equation (14) together with equation (10) as

$$P=\frac{G_{L}f\beta M^{\theta }}{L_{f}}$$ (15)

In case where*f*≤*L*, *L*_*f*_ =*Lf* /365. Substitution of this into equation (15) gives:

$$P=\frac{365G_{L}}{L}\beta M^{\theta }$$ (16)

This equation shows that annual production is the product of leaf biomass and the annualized proportion of the lifetime production of a leaf. In the case where *f* > *L*, *L*_*f*_ = *L* and then:

$$P=\frac{G_{L}}{L}f\beta M^{\theta }$$ (17)

This is also the annualized lifetime production in the case of deciduous plants in a seasonal environment. 

### Toward a Stand Level Production Model

#### Monospecific stand

When a single species constitutes a stand *L*, *f*, β, and *C* in equation (12) can be considered common to all individuals in the stand. In a mono-specific stand, the cumulative number (*N*, m^-2^) and cumulative mass (*Y*, g m^-2^) from the largest to smallest individual is described by the following equation [17] 

$$Y=\frac{N}{JN+K}$$ (18)

where *J* and *K* are parameters specific to a stand and time. In this case, the distribution density function (*ϕ*) of individual plant mass is given by the following equation [17].

$$\phi (M)=\frac{\sqrt{K}}{2J}M^{\frac{-3}{2}}$$ (19)

The total stand level production (*P*_*T*,_ g m^-2^ year^-1^ ) composed of a single species can be expressed by the equation:

$$P_{T}=\int_{M_{\min}}^{M_{\max}}\phi PdM$$ (20)

Substitution of equations (12) and (19) into (20) gives:

$$P_{T}=\frac{3\sqrt{K}}{J(2\theta -1)}\frac{fC}{L}\beta (M_{\max}^{\theta -\frac{1}{2}}-M_{\min}^{\theta -\frac{1}{2}})$$ (21)

If *M*_*min*_ is near zero or far smaller than *M*_*max*_, then the total primary production of a mono-specific stand will be:

$$P_{T}=\frac{3\sqrt{K}}{J(2\theta -1)}\frac{fC}{L}\beta M_{\max}^{\theta -\frac{1}{2}}$$ (22)

#### Mixed-species stand

In a mixed-species stand, species will differ in both their relative abundance and leaf longevity, which complicates the prediction of primary production at the stand level. The primary production (*P*_*i,j*_) for an individual of species *i* can be given by equation (12). The distribution density function of plant mass for each species in the stand in turn is described by *ϕ*_i_. Thus, total primary production of species *i* in the stand is (*P*_*Ti*_) expressed as:

$$P_{Ti}=\int_{M_{\min i}}^{M_{\max i}}\phi_{i}P_{i}dM$$ (23)

where *M*_*maxi*_ and *M*_*mini*_ are, respectively, the maximum and minimum plant weight (g plant^-1^) for each species in the stand. The total production combining all the species in the stand then is expressed by the following equation:

$$P_{Ti}=\sum_{i=1}^{s}\int_{M_{\min i}}^{M_{\max i}}\phi_{i}P_{i}dM$$ (24)

where *s* is the number of species in the stand. When *M*_*mini*_ is small, the value of *P*_*T*_ can be approximated as:

$$P_{Ti}=\sum_{i=1}^{s}\int_{0}^{M_{\max i}}\phi_{i}P_{i}dM$$ (25)

## Discussion

Primary production generally is expressed as some function of the amount of leaves and their efficiency, which is consistent with the approach that Enquist and colleagues adopted in developing a trait-based normalization for prediction of primary production in metabolic scaling theory [4]. Drawing on an assumption that the rate of biomass production for a plant is simply the sum of the production by each of its leaves and building on a classic growth analysis (RGR=NAR·*aL*/*M*_*L*_·*ML*/*M* where *NAR* is net assimilation rate), Enquist and colleagues (*cf* [4] supplemental material, equation S20) derived a generalized model for the annual rate of plant production, *M*_A_: 

$$M_{A}\approx \{[\left(c/\omega \right)A_{L}][a_{L}/m_{L}]\beta \}fM^{\theta }$$ (26)

where *c* is the carbon use efficiency, ω the carbon fraction of a plant, *A*_*L*_ the area-specific carbon assimilation rate of a leaf, *a*_*L*_ the laminar area of a leaf, *m*_*L*_ the mass of a leaf. This model is appropriately framed at the level of the whole plant, avoiding the spurious correlations implicated in leaf level traits influencing productivity [18]. It is noteworthy that the last two terms define the leaf mass per area (LMA), a foliar trait that is proportionate to leaf longevity [16,19] without being subject to spurious correlation [18]. This function for *M*_*A*_ follows the basic allometric model (eq 1) only adjusting the *Y*_*0*_ normalization to the whole plant level {bracketed terms} and annualizing (f) the instantaneous rate of productivity. The derivation is a significant advance in that it reveals the functional traits contributing to the allometric normalization, not only implicating well-recognized roles of traits such as *LMA*, *NAR* and leaf mass ratio as critical elements in plant production but also identifying key roles for carbon use efficiency, carbon fraction, and the allometric coefficient itself. 

On the other hand, the situation is complicated by the difficulties of integrating instantaneous rate of productivity over a year, an integration that is reflected in leaf longevity [5,7]. The valuable insights and conceptual framework provided in equation (26) notwithstanding, it is worthwhile considering whether a simpler expression for *Y*_*o*_ might exist to normalize allometric estimates of primary production. Note that equation (26) is essentially similar to equation (2). From equation (2) we can derive the equation:

$$M_{A}=k\bar{p(t)}fM_{L}$$ (27)

Where, *k* is a constant to convert the amount of carbon assimilated to plant tissue mass (equivalent to *c*/ω), the average daily photosynthetic capacity $\bar{p(t)}$is equivalent to *A*_*L*_(*aL*/*m*_*L*_). When we use an instantaneous assimilation rate of carbon, *P*=*MA*/*k* is expressed by equation (2). The analysis by Enquist and colleagues [4] implicated six traits as critical determinants of the normalization constant for scaling plant primary production. Alternatively, we have shown that primary production can be expressed simply as a function of the amount of leaves and the inverse of leaf longevity (cf. equation 12). If we compare plants of the same size that differ in leaf-longevity, then the primary production of plants with shorter leaf-longevity will be greater than those with longer leaf-longevity. This expectation is supported by a study of seedlings [14]. 

The viability of this simplification ultimately is based on the intrinsic nature of leaf longevity as a trait that integrates the instantaneous processes of plant production over a prolonged time period in which environmental conditions regulating production processes are inevitably varying [12]. This integrative nature of leaf longevity is expressed in the hypothesis that all leaves have a constant net lifetime gain of carbon [5], and hence leaf longevity provides an index of the net outcome of the myriad interactions among functional traits that comprise the adaptation of a species to the environment in which it is growing. It is noteworthy that in this formulation the assimilation rate does not appear to play a role in the normalization constant of production; instead the inverse of leaf longevity acts as an index for the efficiency of photosynthesis integrated over leaf lifetime. The inverse of leaf longevity essentially is equivalent to photosynthetic capacity in equation (12). In general, it appears that leaf longevity can be viewed as a cardinal trait that provides a critical bridge between processes at the tissue and organ levels within a plant and aspects of whole-plant function such as productivity.

### Stand level productivity

The prediction of stand level productivity becomes more difficult because individuals within the stand vary in size even in a relatively simple mono-specific stand such as a field crop or tree plantation. Stand productivity is a function of individual plant mass operating through the frequency distribution of plant mass within a stand. Hozumi and colleagues [17] proposed equation (19) as a globally applicable equation for plant mass and number in vegetation, a relationship that has an ecological basis in logistic growth. Equation (17) includes the plant mass for which distribution density is shown by equation (19), *M* ^-3/2^=*M* ^-12/8^. Enquist [20] also derived a globally applicable distribution of tree diameter (*D*) within a stand as D^-2^, which includes a slightly different distribution function for tree mass (*M* ^-11/8^). Using the distribution function of plant mass in equation (19), we derive equation (21) to predict productivity in a mono-specific stand. Equation (21) shows that stand level production scales as the (θ-½)^th^ power of the maximum plant weight in the stand, e.g. when θ is ¾, the scaling exponent at the stand-level is ¼. Since stand level productivity is considered invariant after canopy closure [5,21], it is curious that the stand productivity increases with the size of the largest plant in the stand. It would seem that there should be mechanisms to damp out unlimited increase in size of the largest plant, and in fact this is the case [22].

As for productivity in mixed-species stands, we have presented a theoretical equation that in principle can predict productivity, but only if data for leaf longevity and size frequency distribution of each species in the stand are available. A less data intensive solution would be desirable. We might consider the possibility that the community-weighted mean of leaf longevity could serve as a normalization constant in estimates of productivity for mixed-species plant communities. In that case tabulated data for the leaf longevity of species could be combined with standard forest inventory data to estimate productivity. This and other possibilities merit consideration.
